# Immunostimulatory Potential of Extracellular Vesicles Isolated from an Edible Plant, *Petasites japonicus*, via the Induction of Murine Dendritic Cell Maturation

**DOI:** 10.3390/ijms221910634

**Published:** 2021-09-30

**Authors:** Jeong Moo Han, Ha-Yeon Song, Seung-Taik Lim, Kwang-Il Kim, Ho Seong Seo, Eui-Baek Byun

**Affiliations:** 1 Advanced Radiation Technology Institute, Korea Atomic Energy Research Institute, Jeongeup 56212, Korea; jmhahn@kaeri.re.kr (J.M.H.); songhy@kaeri.re.kr (H.-Y.S.); ki5336@kaeri.re.kr (K.-I.K.); hoseongseo@kaeri.re.kr (H.S.S.); 2 Department of Biotechnology, College of Life Science and Biotechnology, Korea University, Seoul 02841, Korea; limst@korea.ac.kr; 3 Department of Radiation Science and Technology, University of Science and Technology, Daejeon 34113, Korea

**Keywords:** extracellular vesicles, dendritic cells, immunostimulatory, T cells, *Petasites japonicus*

## Abstract

Extracellular vesicles (EVs) have recently been isolated from different plants. Plant-derived EVs have been proposed as potent therapeutics and drug-delivery nanoplatforms for delivering biomolecules, including proteins, RNAs, DNAs, and lipids. Herein, *Petasites japonicus*-derived EVs (PJ-EVs) were isolated through a series of centrifugation steps and characterized using dynamic light scattering and transmission electron microscopy. Immunomodulatory effects of PJ-EVs were assessed using dendritic cells (DCs). PJ-EVs exhibited a spherical morphology with an average size of 122.6 nm. They induced the maturation of DCs via an increase in the expression of surface molecules (CD80, CD86, MHC-I, and MHC-II), production of Th1-polarizing cytokines (TNF-α and IL-12p70), and antigen-presenting ability; however, they reduced the antigen-uptake ability. Furthermore, maturation of DCs induced by PJ-EVs was dependent on the activation and phosphorylation of MAPK and NF-κB signal pathways. Notably, PJ-EV-treated DCs strongly induced the proliferation and differentiation of naïve T cells toward Th1-type T cells and cytotoxic CD8^+^ T cells along with robust secretion of IFN-γ and IL-2. In conclusion, our study indicates that PJ-EVs can be potent immunostimulatory candidates with an ability of strongly inducing the maturation of DCs.

## 1. Introduction

Extracellular vesicles (EVs) are lipid membrane-enclosed nanoscale vesicles released by most cells and contain protein, lipids, metabolites, and nucleic acids of the source cell [1,2,3]. EVs play crucial roles in cell-to-cell communication as well as in interspecies communication, delivering cargo from donor to recipient cells, which ultimately maintain the physiological conditions [4,5,6]. Based on their functions in transferring bioactive components, EVs are highlighted as potential therapeutic agents that can be used for activities such as immune modulation, drug delivery, and diagnosis [1,7]. In the last decades, there have been several reports on EVs derived from mammalian cells, and recently, there has been an increase in research on plant-derived EVs [8,9,10].

Plant-derived EVs, which have properties similar to those of mammalian EVs, act as extracellular messengers, and perform biological functions, such as anti-inflammatory and antitumor activities, and immunomodulation [11,12]. For example, grape-derived EVs promote the proliferation of Lgr5hi intestinal stem cells via the Wnt/β-catenin pathway in vitro, and oral administration of grape-derived EVs has potential for protecting mice from dextran sulfate sodium-induced colitis [13]. Oral administration of ginger-derived EVs increased the proliferation and survival of intestinal epithelial cells, increased the levels of anti-inflammatory cytokines (IL-10 and IL-22) and reduced those of the proinflammatory cytokines (TNF-α, IL-6, and IL-1β) in colitis models [14]. Lemon-derived EVs had an antitumor effect by inhibiting the proliferation of cancer cells and suppressing tumor growth via the activation of TRAIL-mediated apoptotic cell death [15,16]. Ginseng-derived EVs exert anti-tumor effects by altering M2 polarized macrophages to M1 polarized macrophages via the TLR4 and Myd88 signaling in vitro and in vivo [17]. All the above-mentioned findings provide novel insights highlighting the therapeutic potential of plant-derived EVs [18,19].

*Petasites japonicus* (PJ), known as Butterbur, has been cultivated for hundreds of years in Eastern Asia and is used for dietary and medicinal purposes [20,21,22]. Previous reports have shown that owing to its biological activities, PJ exhibits wide-ranging beneficial therapeutic effects including anticancer [22,23], antiallergy [24], anti-inflammatory [25], and antioxidative activities [26]. Although the antitumor ability of compounds and/or extracts from PJ has been investigated [20,22,23,27,28], the specific immunological role of PJ-EVs has not yet been established.

The present study was aimed at isolating and characterizing plant-derived EVs from PJ leaves, as well as elucidating the immunostimulatory effect of PJ-EVs on dendritic cells (DCs), which have critical roles in the initiation and modulation of innate and adaptive immunity [29].

## 2. Results

### 2.1. Isolation and Characterization of EVs Derived from Petasites japonicus

**Figure 1:**
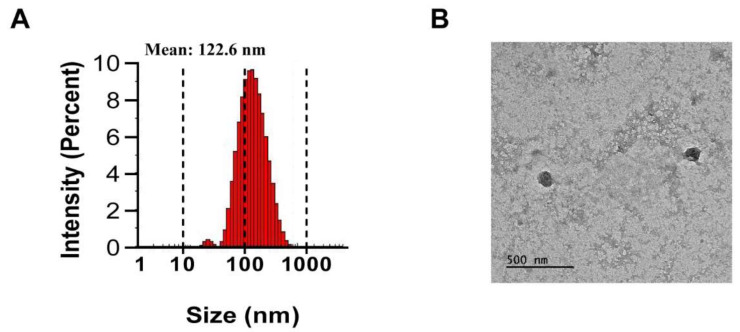
Isolation and characterization of extracellular vesicles (PJ-EVs) derived from the leaves of *Petasites japonicus*. (**A**) Size distribution of PJ-EVs was determined by dynamic light scattering (DLS) analysis. The graph shows results obtained in the with DLS analysis. (**B**) Morphology of PJ-EVs visualized by transmission electron microscopy (TEM). Scale bar = 500 nm.

PJ-EVs were isolated from harvested PJ leaves by filtration and differential centrifugation. The average diameter of PJ-EVs was found to be 122.6 nm by DLS analysis (Figure 1A). The size of PJ-EVs, as determined using TEM analysis, ranged from 100 to 140 nm (Figure 1B) and was in agreement with the DLS results.

### 2.2. PJ-EVs Promote the Expression of Surface Molecules and Production of Th1-Polarizing Pro-Inflammatory Cytokines in BMDCs

**Figure 2:**
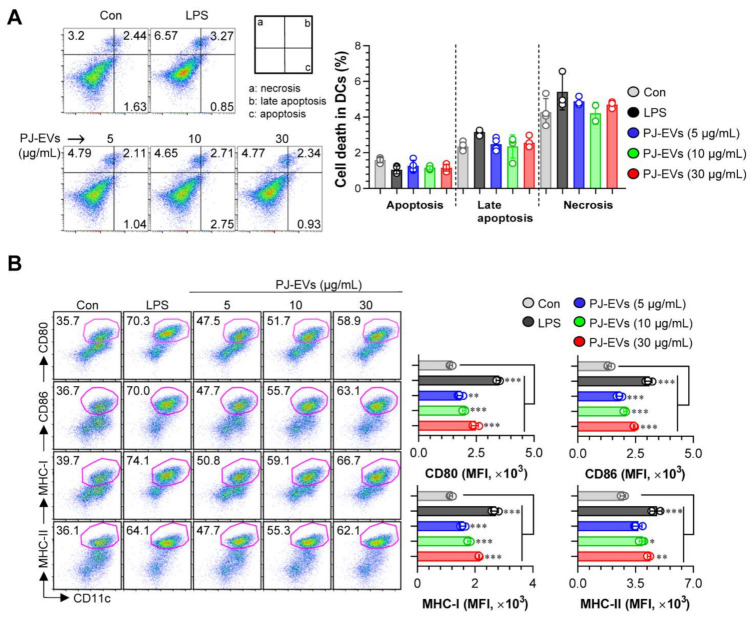
Cytotoxic activity and expression of surface molecules in dendritic cells (DCs) treated with PJ-EVs. (**A**) Cytotoxicity of PJ-EVs in DCs assessed using AnnexinV/propidium iodide (PI) staining (PI^+^ cells: necrosis; AnnexinV^+^PI^+^ cells, early necrosis; AnnexinV^+^ cells, apoptosis). (**B**) Expression of CD80, CD86, MHC-I, and MHC-II in CD11c^+^-gated cells measured by FACS. The percentage and mean fluorescence intensity of surface molecules in CD11c^+^ cells are shown in each panel. * *p* < 0.05, ** *p* < 0.01, or *** *p* < 0.001. SD: standard deviation.

**Figure 3:**
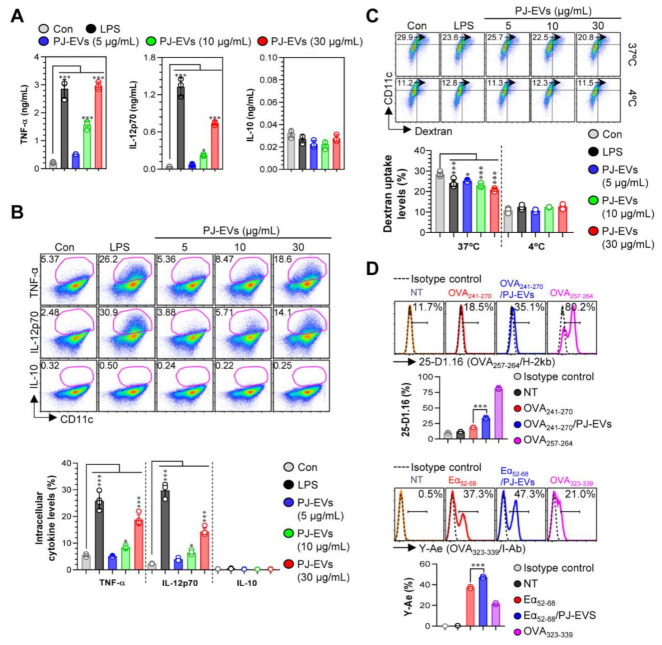
Cytokine production and antigen-uptake and antigen-presenting abilities of DCs stimulated with PJ-EVs. (**A**) Levels of TNF-α, IL-12p70, and IL-10 in the culture supernatant were analyzed using ELISA. (**B**) Intracellular cytokine levels of pro- (TNF-α and IL-12p70) and anti-inflammatory (IL-10) cytokines in untreated and lipopolysaccharide (LPS)- or PJ-EV-treated DCs. The mean fluorescence intensity of intracellular cytokines in CD11c^+^ cells is shown in each panel. (**C**) DCs were stimulated with PJ-EVs or LPS for 18 h and cultured with dextran at 37 or 4 °C for 30 min. These cells were labeled with anti-CD11c antibody and subjected to FACS analysis for detection of dextran uptake. (**D**) Untreated or LPS and PJ-EV-treated cells were treated with OVA_241–270_ (10 μg/mL) or Eα_52–68_ (20 μg/mL) for 24 h. After incubation, each cell was labeled with anti-CD11c, anti-25-D1.16, or anti-Y-Ae mAbs for 15 min. Positive controls for antigen presentation, OVA_257–264_ (5 μg/mL) or OVA_323–339_ (5 μg/mL), were used. Histogram data and bar graphs show the expression of the OVA_257–264_/H-2Kb and OVA_323–339_/I-Ab in the gated CD11c^+^ population. Bar graph data are shown as the mean ± SD (n = 3 samples) of three representative experiments. * *p* < 0.05, or *** *p* < 0.001. SD: standard deviation.

DCs are professional Ag-presenting cells (APCs) that induce the differentiation of naïve T cells into memory and effector T cells. Importantly, matured DCs exhibit phenotypic alterations, including the enhanced expression of surface molecules, production of cytokines, Ag-presenting ability as well as decreased Ag-uptake ability [30,31]. Based on the features of DCs, in the present study, we examined whether treatment with PJ-EVs induces the maturation and activation of BMDCs. To determine the maximum threshold of PJ-EVs for all experiments, the effect of PJ-EVs on the proliferation rate of BMDCs was investigated. As shown in Figure 2A, PJ-EVs showed no cytotoxicity at concentrations below 30 μg/mL. Next, to investigate the expression of surface molecules, BMDCs were treated with LPS (positive control for maturation of DCs) and PJ-EVs (5, 10, and 30 μg/mL). PJ-EVs significantly increased the expression of surface molecules, such as CD80, CD86, MHC-I, and MHC-II on BMDCs in a dose-dependent manner (Figure 2B). Additionally, PJ-EV treatment considerably enhanced the production of extracellular cytokines, TNF-α and IL-12p70, which are characterized as the Th1-polarizing proinflammatory cytokines in BMDCs. However, PJ-EVs did not increase the levels of IL-10, which is characterized as an anti-inflammatory and immunosuppressive cytokine in BMDCs (Figure 3A). These results were consistent with the levels of intracellular cytokines on PJ-EV-treated BMDCs determined using flow cytometry (Figure 3B).

### 2.3. Effects of PJ-EVs on Ag-Uptake and Ag-Presenting Abilities of BMDCs

Upon maturation, the ability of DCs to uptake antigens decreases and their ability to present antigens increases [32]. As shown in Figure 3C, LPS (positive control)-, and PJ-EV-treated BMDCs displayed significantly reduced cellular uptake levels of dextran (an antigen) compared with untreated BMDCs. Next, we determined the Ag-presenting ability of PJ-EV-treated BMDCs using the anti-25D1.16 mAb, which responds to the OVA_257–264_ peptide that complexes with the H-2Kb of MHC-I, and anti-Y-Ae mAb, which directly combines with the OVA_323–339_-MHC-II complex. OVA_257–264_ and OVA_323–339_ peptides were used as positive controls for the presentation of MHC-I and MHC-II. As shown in Figure 3D, significantly increased percentage of the OVA_257–264_/MHC-I and OVA_323–339_/MHC-II complexes were detected in PJ-EV-treated DCs. Taken together, these results suggest that PJ-EVs strongly induced the maturation of DCs by increasing the production of Th-1-polarizing cytokine, expression of surface molecules, and Ag-presenting ability and reducing the Ag- uptake ability.

### 2.4. PJ-EVs Induce the Maturation of DCs via the Activation of MAPK and NF-ĸB Signaling Pathways

**Figure 4:**
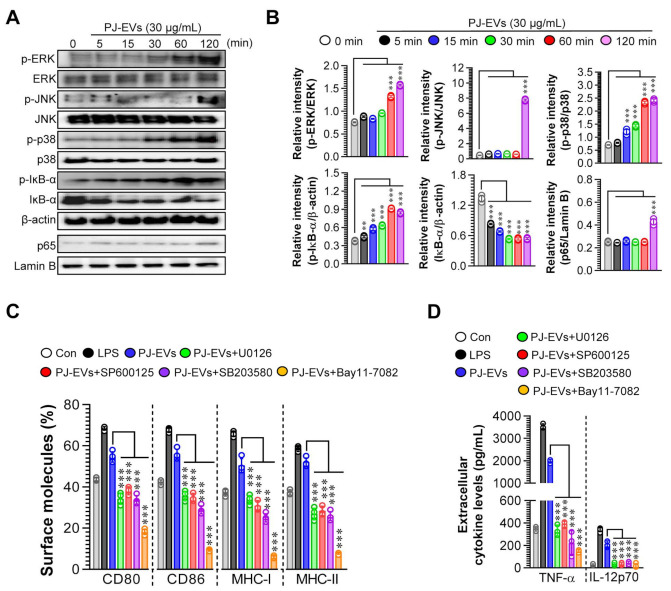
Activation of MAPK and NF-κB signaling pathways in DCs treated with PJ-EVs. (**A**) DCs were incubated with PJ-EVs (30 μg/mL) for different times. After incubation, these cells were lysed for protein extraction. SDS-PAGE and immunoblotting analyses were performed to assess the maturation signal using phosphorylated antigen-specific and total antigen-specific antibodies against ERK, JNK, p38, IĸB-α, and NF-κB. Data are representative of three independent experiments. (**B**) Western blotting data were assessed using the Image J software to compare the phosphorylated and total forms. (**C**,**D**) DCs pretreated with MAPK and NF-κB signaling pathway inhibitors, U0126 (ERK inhibitor, 10 μM), SP600125 (JNK inhibitor, 20 μM), SB203580 (p38 inhibitor, 20 μM), and Bay11-7082 (NF-κB inhibitor, 10 μM), were incubated with PJ-EVs (30 μg/mL) for 18 h. (**C**) Expression levels of surface molecules (CD80, CD86, MHC-I, and MHC-II) on cells analyzed by FACS. (**D**) Levels of extracellular cytokines (TNF-α and IL-12p70) were analyzed using ELISA. The results are indicated as mean ± SD (n = 4 samples) of three representative experiments. ** *p* < 0.01, or *** *p* < 0.001. SD: standard deviation.

The activation of MAPK and NF-ĸB signaling pathways is important for inducing the maturation of DCs [33]. Thus, we investigated whether the maturation of DCs induced by PJ-EVs needed the activation of MAPK and NF-ĸB signaling pathways. As shown in Figure 4A,B, PJ-EVs initiated the activation of MAPK and NF-ĸB signaling pathways via the phosphorylation of MAPKs (ERK, JNK, and p38), phosphorylation of IĸB, and nuclear translocation of p65 in BMDCs. In addition, to confirm whether phenotypic alterations in mature DCs induced by PJ-EVs require the activation of MAPKs and NF-ĸB signals, we determined the expression of surface molecules and production of cytokines in PJ-EV-treated DCs in the presence and absence of MAPK and NF-ĸB inhibitors. In the presence of these inhibitors, the expression of surface molecules (Figure 4C; CD80, CD86, MHC-I, and MHC-II) and production of cytokines (Figure 4D; TNF-α, IL-12p70) were significantly reduced in PJ-EV-treated DCs, suggesting that MAPK and NF-ĸB signaling pathways are crucial for PJ-EV-induced maturation of DCs.

### 2.5. PJ-EV-Stimulated DCs Induce Naïve T Cells toward Th1 Polarization and Activated CD8+ T Cells

**Figure 5:**
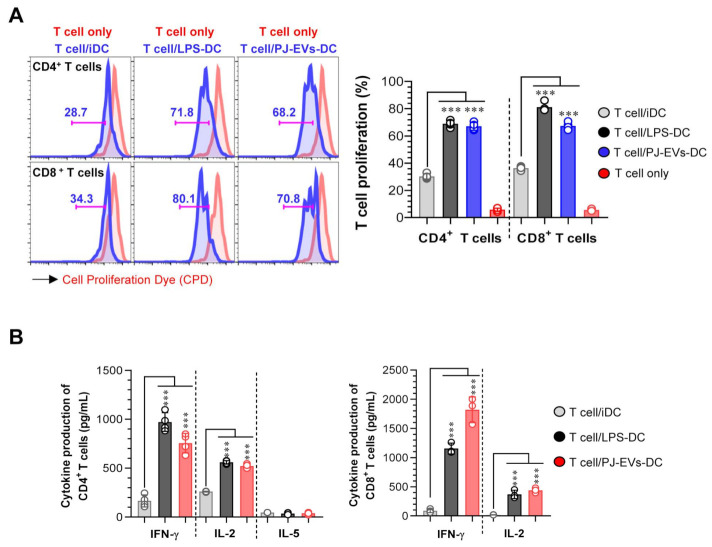
DCs treated with PJ-EVs initiate T cell proliferation and Th1 response. (**A**) PJ-EVs (30 μg/mL)-treated DCs were cocultured with CPD-stained BALB/c background CD4^+^ and CD8^+^ T cells at a ratio of 0.2:1 (DCs and T cells, respectively). After 2 days of coculture, the proliferation of CD4^+^ or CD8^+^ T cells was analyzed by FACS. (**B**) Levels of indicated cytokines in the culture supernatant were analyzed using ELISA. The results are indicated as mean ± SD (n = 4 samples) of three representative experiments. *** *p* < 0.001. SD: standard deviation.

To demonstrate the interaction between PJ-EV-treated DCs and T cells, allogeneic MLR was performed. PJ-EV-treated DCs were cocultured with CPD-labeled allogeneic CD4^+^ or CD8^+^ T cells isolated from BALB/c mice for 2 days to investigate whether PJ-EV-treated DCs could initiate T-cell proliferation. Notably, the proliferation of CD4^+^ and CD8+ T cells was induced in the PJ-EV-treated DCs compared with that in non-treated DCs (Figure 5A). Additionally, the production of cytokines from CD4^+^ and CD8^+^ T cells cocultured with untreated or LPS- and PJ-EV-treated DCs in the culture supernatant was measured. IFN-γ and IL-2 are cytokines characteristic of Th1 and activated CD8^+^ T cells, and IL-5 is cytokine produced by Th2 [34]. As shown in Figure 5B, CD4+ and CD8+ T cells cocultured with PJ-EV-treated DCs produced higher amounts of IFN-γ and IL-2 compared with CD4+ and CD8+ T cells cocultured with untreated DCs. In contrast, CD4+ T cells co-incubated with PJ-EV-treated DCs secreted significantly lower levels of IL-5.

## 3. Discussion

*Petasites japonicus*, consumed as a dietary plant in China, Japan, and Korea, has been reported for multiple beneficial effects, such as anti-inflammatory, antioxidant, antiallergic, and anticancer properties [21,22,25]. Considering these beneficial properties, in the present study, we successfully isolated and analyzed EVs from leaves of PJ. In addition, we investigated the immunological functions of PJ-EVs by assessing their ability to activate DCs and induce their maturation by increasing the co-stimulation of surface molecules, enhancing their ability of Ag presentation and decreasing their ability of Ag uptake, and secretion of Th1-polarizing proinflammatory cytokines via the activation of MAPK and NF-ĸB signaling pathways. Notably, PJ-EV-induced maturation of DCs initiated naïve T cells to polarize CD4^+^ and activated CD8^+^ T cells along with the production of IFN-γ and IL-2. To the best of our knowledge, this is the first study to suggest that EVs derived from an edible plant, PJ, possess immunostimulatory effects and induce the maturation of DCs.

DCs are known as professional APCs and play a critical role in initiating and modulating the innate and adaptive immunity [35]. Immature DCs are characterized by low expression of cell surface molecules and cytokines as well as high endocytic activity. On the contrary, maturation of DCs, induced by external antigens or damage-associated molecular patterns, is followed by upregulation of MHCs associated with the antigen (signal 1), costimulatory molecules such as CD80 and CD86 (signal 2), and proinflammatory cytokines, including IL-12 and TNF-α (signal 3). Based on the phenotypic alterations in DCs, they can induce the activation and proliferation of T lymphocytes [36,37] In this respect, strong induction of DC maturation can initiate Th1 polarization and CD8^+^ T cell activation and has potential for the initiation of anticancer immunity [38,39]

Our data demonstrate that PJ-EVs significantly induce phenotypic and functional change in DC maturation. PJ-EVs treated DCs induced an increase in the expression of co-stimulatory molecules (CD80 and CD86) as well as MHC-I and MHC-II, which have crucial roles in interaction between DCs and T cells [31]. In addition, PJ-EVs induced a significant increase in the production of TNF-α and IL-12 which were characterized by the Th1-cytokines, however, did not induce the production of the IL-10 which were characterized by Th2-cytokines in DCs. These results suggest that PJ-EV-treated DCs produce increasingly amounts of IL-12, the key factor supporting Th1 polarization and CTL response, which could have potent anticancer effects [40]. Furthermore, when the maturation of DCs proceeds, the Ag-presenting ability is increased, whereas Ag-phagocytic ability is decreased. Our data suggest that PJ-EVs induced an increase in the Ag-presenting abilities and diminished the Ag-uptake ability of DCs. These results indicate the presence of typical features in DCs matured by treatment with PJ-EVs [41,42]. The MAPK and NF-ĸB signaling pathways are involved in the regulation of phenotypic and functional maturation of DCs [43,44]. We found that the PJ-EV-induced maturation of DCs was dependent on the activation and phosphorylation of the MAPK and NF-ĸB signaling pathways. These data suggest that PJ-EVs can strongly induce the maturation DCs. Based on these results, we further performed the MLR assay for elucidating the potential of PJ-EVs in enhancing the anticancer immunity. Allogeneic coculture of PJ-EV-treated DCs with naïve CD4^+^ and CD8^+^ T cells initiated the proliferation and activation of the CD4^+^ and CD8^+^ T cells along with significantly secretion of IFN-γ and IL-2. Given the fact that polarization of Th1 and activation of CD8^+^ T cells as well as the robust production of Th1 cytokines, such as IFN-γ and IL-2, is important for inducing anticancer immunity, our results demonstrate that PJ-EVs can be potent immunostimulatory candidates [45,46]. Several clinical trials were reported using the plant-derived EVs, such as grape, ginger, and aloe [1]. Considering the potent immunostimulatory properties of PJ-EVs, we have planned for assessing the clinical trials using PJ-EVs after further studies in animal models. These clinical studies may be anticipated results because of the immunostimulatory effect of PJ-EVs as well as the biocompatible properties of EVs [7].

## 4. Materials and Methods

### 4.1. Ethics Statement and Mice

Seven to eight-week-old C57BL/6 and BALB/c female mice purchased from Orient Bio Inc. (Seoul, Korea) were used for experiments on differentiation of bone marrow-derived dendritic cells (BMDCs) and for isolation of splenocytes. All animal experiments were assessed according to the established guidelines of Korea Atomic Energy Research Institute (KAERI, Jeongeup, Korea) and approved by the Institutional Animal Care and Use Committee (IACUC) of KAERI (permit number: KAERI-IACUC-2020-002).

### 4.2. Isolation and Characterization of PJ-EVs

For isolation of PJ-EVs, fresh PJ leaves were purchased from the local grocery market (Jeongeup-si, Jeollabuk-do, Korea) and washed three times with distilled water at room temperature (RT; 20 °C). After the final wash, PJ leaves were ground in a slow juicer to obtain PJ juice, which was sequentially centrifuged at 200× *g* for 10 min, 2000× *g* for 20 min, and 10,000× *g* for 30 min to remove large particles and fibers. The final supernatant was filtered using a 0.22 μm bottle-top vacuum filter system (Corning, Merck KGaA, Darmstadt, Germany) and the filtrate was ultracentrifuged at 100,000× *g* for 60 min (Beckman Optima XE-100, Beckman, Indianapolis, IN, USA). The pellet obtained after ultracentrifugation was resuspended in phosphate-buffered saline (PBS) and the solution was filtered (through a 0.22 μm filter) and stored at −80 °C until further use. The hydrodynamic size of PJ-EVs was analyzed by dynamic light scattering (DLS) using a Zetasizer nano ZS Zen3600 (Malvern, UK). For transmission electron microscopy (TEM), samples were dispersed in ethanol, and the dispersed solution was mounted onto a carbon support film on 150 mesh nickel grid and dried. Observations were made using a field emission transmission electron microscope (FE-TEM) with JEM-2100F (JEOL Ltd., Tokyo, Japan) at an acceleration voltage of 200 KV. The protein content in PJ-EVs was measured using a BCA protein assay kit (Thermo Scientific Pierce, Rockford, IL, USA) following the manufacturer’s instructions.

### 4.3. Antibodies and Reagents

Primary cell culture was performed for differentiation of BMDCs using recombinant mouse (rm)-granulocyte-macrophage colony-stimulating factor (rm-GM-CSF) and rm-interleukin-4 (rm-IL-4) from JW CreaGene (Daegu, Korea). Ultrapure lipopolysaccharide (LPS) from Escherichia coli serotype 0111:B4 (Invivogen, San Diego, CA, USA) was used as a positive control for in vitro maturation of BMDCs. For assessment of cytotoxicity, Annexin V and propidium iodide (PI) antibodies (Abs) were purchased from BD Bioscience (San Jose, CA, USA). The maturation, activation, and phenotypic alteration of DCs and T cells were analyzed by flow cytometry (FACSverse, BD Bioscience, Bergen County, NJ, USA) using the following fluorescence-conjugated Abs: Live/Dead Cell Staining Kit (L/D; BV510) from Invitrogen (Carlsbad, CA, USA); Cell Proliferation Dye (CPD) eFluor 450, anti-MHC-I (APC), -MHC-II (PE-Cy7), -CD3 (APC-Cy7), -CD4 (Alexa488), -CD8α (Percp-Cy5.5), -IFN-γ (PE), -TNF-α (APC), and -IL-2 (PE-Cy7) Abs from eBioscience (San Diego, CA, USA); anti-CD11c (PE-Cy7), -CD80 (FITC), -CD86 (PE), -TNF-α (APC), -IL-12p70 (PE), and -IL-10 (FITC) Abs from BD Bioscience. The controls for the expression of surface molecules on DC were the corresponding isotype Abs for surface molecules, namely rat IgG2 kappa (FITC), rat IgG2a kappa (PE), rat IgG2a kappa (APC), and rat IgG2b kappa (PE-Cy7) from BD Bioscience. The measurement of extracellular cytokines was performed via enzyme-linked immunosorbent assay (ELISA) using the following mouse-specific ELISA kits: TNF-α, IL-12p70, IL-10, IFN-γ, IL-5, and IL-2 ELISA kits from BD Bioscience. The antigen (Ag)-uptake ability was analyzed using FITC-conjugated dextran (40,000 Da; Sigma, St. Louis, Mo, USA). The Ag-presenting ability was assessed using OVA_241–270_, Eα_52–68_, OVA_257–264_, and OVA_323–339_ from AbFrontier (Seoul, Korea). MAPK and NF-ĸB signaling pathways were analyzed using the following mouse Abs (mAbs) for immunoblotting and signaling pathway-specific pharmacological inhibitors: phosphorylated (p)-ERK, p-JNK, p-p38, p-IĸB-α, total-ERK, total-JNK, total-p38, IĸB-α, NF-ĸB, lamin B, and β-actin Abs from Cell Signaling Technology (Boston, MA, USA); U0126 (ERK), SP600125 (JNK), SB203580 (p38), and Bay11-7082 (NF-ĸB) from Calbiochem (San Diego, CA, USA). Naive CD4^+^ and CD8^+^ T cells were isolated with the MACS MicroBeads system using CD4^+^ and CD8^+^ isolation kits (Miltenyi Biotec, San Diego, CA, USA).

### 4.4. Treatment of BMDCs with PJ-EVs

For differentiation of BMDCs, bone marrow cells were pumped out from femurs and tibias, and red blood cells were lysed with a red blood cell-lysis buffer (Sigma-Aldrich, St. Louis, MO, USA). The lysed cells were cultured for 8 d in RPMI-1640 medium (Biowest, Nuaille, France) supplemented with 10% heat-inactivated fetal bovine serum (FBS, Biowest), 1% penicillin/streptomycin (P/S, Gibco, Carlsbad, CA, USA), 20 ng/mL GM-CSF, and 0.5 ng/mL IL-4. After 8 days of differentiation, cells were harvested and confirmed for CD11c+ population of BMDCs (>90% purity) by FACSverse using anti-CD11c Ab. For PJ-EV treatment, BMDCs (0.5 × 10^6^/well in a 48-well plate) were treated with a wide range dose (5, 10, and 30 μg/mL) of PJ-EVs and incubated for 18 h at 37 °C/5% CO_2_. LPS (100 ng/mL) was used as a positive control for maturation of DCs.

### 4.5. Annexin V and PI Staining

PJ-EV-treated BMDCs (treated for 18 h) were harvested and stained with Annexin V (diluted at 1:50 in the Annexin V binding buffer, BD Bioscience) for 15 min at RT. The cells were then washed with the Annexin V binding buffer and stained with PI (diluted at 1:25 in the Annexin V binding buffer) for 10 min at RT. Necrotic, late apoptotic, and apoptotic cell death was quantitated by detecting cells positive for Annexin V, PI, or both, respectively, using a FACSverse cytometer and the FlowJo software (V10, BD Bioscience).

### 4.6. Analysis of Surface Molecules on BMDCs

PJ-EV-treated BMDCs (treated for 18 h) were harvested and stained with L/D, anti-CD80, -CD86, -MHC-I, and MHC-II Abs for 20 min at RT. The cells were washed with cold FACS washing buffer (2% FBS and 0.01% sodium azide in PBS), and then fixed with IC fixation buffer (eBioscience). The expression levels of surface molecules on cells were analyzed using the FACSverse cytometer and FlowJo software.

### 4.7. Measurement of Extracellular Cytokine Levels

To determine the levels of extracellular cytokines, supernatants harvested from PJ-EV-treated BMDCs (treated for 18 h) were analyzed using ELISA. All the steps of ELISA were performed in accordance with the manufacturer’s instructions. The levels of immunoreactive cytokines (TNF-α, IL-12p70, and IL-10) were measured by determining the absorbance at 450 nm using a microplate ELISA reader (Zenyth 31004 Anthos Labtec Instruments GmbH, Salzburg, Austria).

### 4.8. Detection of the Levels of Intracellular Cytokines in BMDCs

BMDCs were treated with PJ-EVs in the presence of GolgiPlug (BD Bioscience) for 8 h, and the cultured cells were subsequently collected and washed with cold FACS washing buffer. Next, the harvested cells were stained with L/D and anti-CD11c Ab for 20 min at RT and washed with cold FACS washing buffer. The cells were fixed and permeabilized using BD Cytofix/Cytoperm buffer for 20 min at 4 °C and washed with BD Perm/Wash buffer. Thereafter, the cells were stained with anti-TNF-α, -IL-12p70, and -IL-10 Abs. Following staining, the cells were washed two times with BD Perm/Wash buffer, and levels of intracellular cytokines in BMDCs (CD11c+ cells) were analyzed using the FACSverse cytometer and FlowJo software.

### 4.9. Analysis of the Antigen-Uptake Ability of BMDCs

PJ-EV-treated BMDCs (treated for 18 h) were incubated with 1 mg/mL dextran for 40 min at 37 °C. Cells were washed three times with cold FACS washing buffer and stained with anti-CD11c Ab for 20 min at RT. The cells were then washed with cold FACS washing buffer and the uptake levels of dextran in BMDCs (CD11c+Dextran+ cells) were analyzed using the FACSverse cytometer and FlowJo software.

### 4.10. Analysis of Antigen-Presenting Ability of BMDCs

To investigate the Ag-presenting ability of PJ-EV-treated BMDCs, the peptide-MHC-I and MHC-II complex formation using the OVA_257–264_ and OVA_323–339_ was evaluated. BMDCs were treated with PJ-EVs in the presence of OVA_241–270_ (10 μg/mL, Sigma-Aldrich) for 18 h to assess the formation of peptide-MHC-I complex. BMDCs were treated with PJ-EV in the presence of Eα_52–68_ (20 μg/mL, AbFrontier, Seoul, Korea) for 18 h to analyze the formation of peptide-MHC-II complex. As a positive control, 5 μg/mL OVA_257–264_ and OVA_323–339_ (both from AbFrontier) were used (3 h treatment of BMDCs). The cells were harvested, washed two times with cold FACS buffer, and then stained with anti-CD11c (PE-Cy7), anti-25-D1.16 (eBioscience), or anti-Y-Ae Abs (eBioscience) for 15 min at RT. Thereafter, the cells were washed with FACS washing buffer and the Ag-presenting ability of BMDCs was analyzed using the FACSverse cytometer and FlowJo software.

### 4.11. Western Blotting Analysis

BMDCs were treated with PJ-EVs for different times (0, 5, 15, 30, 60, and 120 min). Cytosolic and nuclear proteins were extracted by cell lysis using a cell lysis buffer (RIPA buffer, Pierce, Rockford, IL, USA) and the CelLytic NuCLEAR Extraction Kit (Sigma-Aldrich), respectively. The subsequent steps were performed as described previously [47].

### 4.12. Assessment of the Induction of Maturation of PJ-EV-Treated BMDCs by Inhibition of the MAPK and NF-ĸB Signaling Pathways

BMDCs were preincubated with MAPK (U0126; 10 μM, SP600125; 20 μM, SB20358; 20 μM), and NF-ĸB (Bay11-7082; 10 μM) signaling inhibitor for 2 h prior to treatment with PJ-EVs for 20 h. After stimulation, cells were analyzed for the expression of surface molecules (CD80, CD86, MHC-I, and MHC-II) of DCs using the FACSverse cytometer, and cell culture supernatant was used to measure the levels of extracellular cytokines (TNF-α and IL-12p70) using ELISA.

### 4.13. Allogeneic Mixed Lymphocyte Reaction

CD4^+^ and CD8^+^ T cells were isolated from the spleen of BALB/c mice using MACS CD4^+^ and CD8^+^ isolation kits. The isolated T cells were stained with 1 μM Cell Proliferation Dye (CPD) eFluor 450 in a 37 °C water bath for 15 min in the dark. The cells were then washed three times with cold PBS containing 10% FBS. Finally, CPD-labeled T cells (5 × 10^5^/well in a 96-well plate) were cocultured with untreated or LPS- and PJ-EV-treated BMDCs (1 × 10^5^/well in a 96-well plate) in the presence of RPMI-1640 medium supplemented with 10% FBS and 1% P/S. After 3 d of culturing, supernatant was collected to measure the levels of extracellular cytokines (IFN-γ, IL-5, and IL-2) using ELISA. Additionally, CD4^+^ and CD8^+^ T cells were harvested and washed twice with cold FACS washing buffer and subsequently stained with anti-CD4 and -CD8 Abs, respectively. Thereafter, cells were washed twice with cold FACS washing buffer and proliferation levels of CD4^+^ and CD8^+^ T cells were analyzed using the FACSverse cytometer and FlowJo software.

### 4.14. Statistical Analysis

Data were analyzed using Tukey’s unpaired t tests or multiple comparison tests with GraphPad Prism 7 (2018, GraphPad, San Diego, CA, USA). Data are represented as the mean ± standard deviation (SD). * *p* < 0.05, ** *p* < 0.01, and *** *p* < 0.001 were considered statistically significant.

## 5. Conclusions

In conclusion, we report for the first time, the immunological roles of EVs derived from PJ in the maturation and activation of DCs. The average diameter of PJ-EVs, as assessed by DLS and TEM, was 122.6 nm. Furthermore, we assessed the immunological activity of PJ-EVs using DCs. PJ-EVs induced the upregulation of CD80, CD86, MHC-I, and MHC-II on DCs and increased the secretion of Th1-polarizing cytokines. In addition, PJ-EVs enhanced the Ag-presenting ability and decreased the Ag-uptake ability of DCs. Interestingly, the induction of the maturation of DCs by PJ-EVs was dependent on the activation and phosphorylation of MAPKs and NF-ĸB signaling pathways. Importantly, PJ-EV-treated DCs triggered the activation of adaptive immune response by differentiation and proliferation of the naïve T cells toward IFN-γ-producing Th1-type T cells and activated the cytotoxic CD8^+^ T cell response. In future research, we would focus on elucidating the anticancer effects of orally administered PJ-EVs on immune cells via in a tumor-bearing mouse model.
